# Presentations Due to Priapism in an Urban Hospital in Switzerland

**DOI:** 10.1155/emmi/9996341

**Published:** 2026-02-19

**Authors:** Julian Dionigi Uhl, Lukas Koneval, Laila Schneidewind, Manuel Haschke, Aristomenis Exadaktylos, Evangelia Liakoni

**Affiliations:** ^1^ Department of Emergency Medicine, Lindenhof Hospital, Lindenhofgruppe, Bern, Switzerland; ^2^ Department of General Internal Medicine, Clinical Pharmacology and Toxicology, Inselspital, Bern University Hospital, University of Bern, Bern, Switzerland, unibe.ch; ^3^ Department of Urology, Inselspital, University Hospital Bern, University of Bern, Bern, Switzerland, unibe.ch; ^4^ Department of Emergency Medicine, Inselspital, University Hospital Bern, University of Bern, Bern, Switzerland, unibe.ch

**Keywords:** adverse effects, drug-induced, emergency medicine, priapism, urology

## Abstract

**Objective:**

Priapism, a persisting erection not associated with sexual stimulation, can be ischaemic, with the risk of permanent erectile dysfunction, or nonischaemic. Drugs—e.g., injection therapies for erectile dysfunction, as well as neuroleptics, antidepressants and various other medicines—can also cause priapism. This study aimed to describe presentations due to priapism and provide insights into specific causes, clinical presentations, diagnostic strategies and emergency management.

**Methods:**

A single‐centre, retrospective, observational study of patients (≥ 16 years old) presenting to the University Hospital of Bern, Switzerland, between January 2010 and June 2023 due to priapism. The cases were retrieved from the electronic health records using full‐text search.

**Results:**

During the study period, 40 cases corresponding to 32 patients were included. The mean ± SD age was 48 ± 15 years, and pain was present in 21 cases (53%) on presentation. Median time of erection was 15 h (range: 1–80, *n* = 23). A penile blood gas analysis was performed in 32 cases (80%), and 29 of these (91%) were of the low‐flow type. Most commonly suspected causes were idiopathic (*n* = 25, 63%) and drug‐induced (*n* = 10, 25%). Suspected agents in the drug‐induced cases were corpus cavernosum autoinjection therapy (*n* = 4), trazodone (*n* = 3), sildenafil (*n* = 2) and urapidil (*n* = 1). Puncture of the corpus cavernosum and injection of noradrenalin and adrenalin were the therapeutic measure in 35 cases (88%). In 13 cases, there was at least one recurrence, including 10 within one week. Drugs given as recurrence prophylaxis included tadalafil (*n* = 9) and diazepam (*n* = 4).

**Conclusion:**

Presentations due to priapism appear to be rare, but the majority of the cases presented with ischaemic priapism, which is a medical emergency. The findings can be used to identify areas requiring further research (e.g., drugs used as recurrence prophylaxis) and raise awareness of this potentially severe complication—which patients are often ashamed to report.

## 1. Introduction

Priapism is defined as a persistent erection (usually defined as lasting longer than 4 h, but definitions vary) that is not associated with sexual stimulation [1]. Priapism is not very common (0.5–0.9 cases per 100,000 person‐years [1]) but can negatively affect quality of life and sexual function [2]. The two main forms are low‐flow (ischaemic) and high‐flow (nonischaemic) priapism [1, 3]. Ischaemic priapism (most common, > 95% of the cases) is caused by venous occlusion that leads to stasis, with subsequent reduced arterial blood flow and, similar to a compartment syndrome, brings a risk of permanent erectile dysfunction due to hypoxia [1, 4, 5]. As a consequence of blood stasis, thrombosis of the corpora cavernosa can occur after some hours, leading to fibrosis, and this form of priapism is thus a medical emergency [1]. Low‐flow priapism can also be associated with sickle cell disease (also described as intermittent or stuttering priapism) [1]. On the other hand, nonischaemic priapism may be caused by, for example, trauma and has a lower risk of hypoxia [1]. It is then very important to distinguish between the two forms, as this can decide the further management. In addition to the patient’s history and physical examination, a blood gas analysis (BGA) of the aspirated corporal blood can also help to distinguish between ischaemic and nonischaemic forms [1, 3].

Possible causes of priapism include haematological diseases (e.g., sickle cell anaemia), surgical interventions, trauma, haematological conditions (e.g., leukaemia), local infiltration by tumours and other compressive causes (e.g., disc hernia), infections, toxins (e.g., cocaine, alcohol, marijuana and spider bites), metabolic diseases (e.g., diabetes mellitus) and medications (e.g., injection therapies for erectile dysfunction, but also oral medications such as neuroleptics, antidepressants and various other drug groups [6, 7]), while priapism can also be idiopathic [1, 3, 4]. Drug‐induced priapism is usually low‐flow and, if a specific drug is identified as a cause, this should be discontinued [1, 4].

In this project, we retrospectively analysed cases presenting to the University Hospital of Bern due to priapism, in order to describe specific causes, clinical presentation, diagnostic strategies and emergency management of such cases. These findings should help to investigate potential risk factors (e.g., specific substances and substance groups) and raise awareness of this medically important but often underreported complication.

## 2. Materials and Methods

A single‐centre, retrospective, observational study including patients ≥ 16 years old presenting to the University Hospital Bern, Switzerland, between January 1 2010 and June 30 2023 due to priapism. The study was approved by the local ethics committee (project ID No. 2023–013759). The data were extracted from the electronic health records by the Insel Data Science Center (IDSC), using the German word for priapism (*Priapismus*) as the search term (free text or coded diagnosis [ICD‐10] containing the word ‘*Priapismus’*). The exact search term was ‘*%priapismus%’,* the % meaning that it could be preceded and followed by any combination of characters. Patients were excluded if they had rejected the hospital’s General Consent for further use of their medical data for research purposes. We also excluded cases with insufficient available information (e.g., patients who left the emergency department before being seen by the medical staff) and patients presenting for a follow‐up after a previous incidence of priapism but without acute complaints at the current presentation.

The following data were extracted, if available, from the electronic health records: age, medications (including dose and duration of therapy), comorbidities, date of presentation, type of admission, symptoms (including duration of priapism), laboratory results (e.g., BGA), diagnostic procedures, treatment, outcome and disposition following the emergency department stay (e.g., discharge or admission to hospital). Priapism was classified as low‐ or high‐flow on the basis of the hospital’s lab report references for the BGA, i.e., for low‐flow priapism: pH < 7.25, partial pressure of oxygen (PO_2_) < 30 mmHg and partial pressure of carbon dioxide (PCO_2_) > 60 mmHg. For patients presenting more than once during the study period, each priapism‐related presentation was treated as a separate case. Priapism‐related presentations were classified as ‘drug‐induced’ if there was at least a possible causal relationship, as based on the reports in the electronic health records. Causality assessment was performed using the Naranjo Adverse Drug Reaction Probability Scale [8]. We recorded data using Microsoft Excel. Results are presented descriptively as counts and percentages or median and range or mean and standard deviation, as appropriate.

## 3. Results

During the study period, a total of 53 patients and 163 cases were identified and extracted by the IDSC. Nineteen cases were excluded because of lack of consent for data use, and one because the patient was younger than 16 years. A further 103 cases were excluded because the presentation was not related to priapism or the priapism was in the past without current acute complaints (99 cases), or priapism was described as suspected but without a definitive diagnosis (4 cases). Finally, 40 cases from 32 patients were included in the analysis (four patients presented more than once: two patients with 4 cases each and two patients with 2 cases each).

The mean ± SD age of the patients on first presentation was 48 ± 15 years (range 20–73). Most cases (*n* = 36, 90%) presented via the emergency department, three cases (8%) via the outpatient clinic of the urology department, and in one case (2%) the route of admission was unknown. Sixteen presentations (40%) occurred during springtime, with a peak in April (*n* = 8, 20%). Figure 1 shows the annual and monthly distribution of the included cases.

FIGURE 1Monthly (a) and annual (b) distribution of the included cases (*N* = 40; for patients presenting more than once, each case was counted separately).

**Figure:**
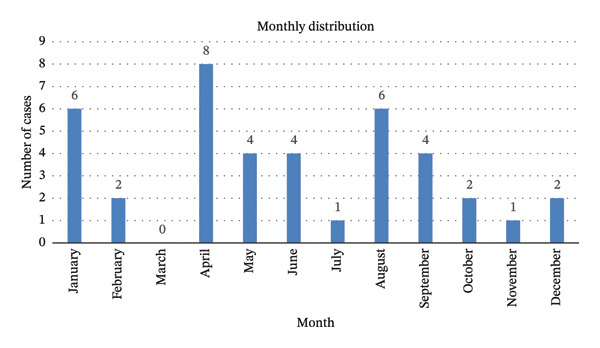
(a)

**Figure:**
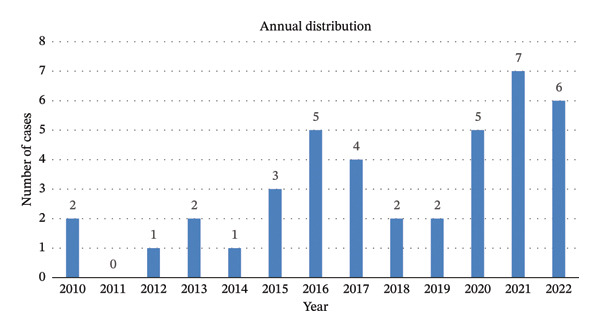
(b)

In 29 of the 40 cases (73%), the entry medication was documented on presentation, with an average of 4 (range 1–14) medications per case. The most frequent classes of medication were psychotropics (*n* = 9), analgesics (*n* = 7), antiepileptics (*n* = 6), anti‐infectives (*n* = 6) and benzodiazepines (*n* = 5). Out of 23 cases (58%) with available information, the median time of erection was 15 h (range 1–80 h), while in 17 cases (43%) this information was not documented (among those, 6 [35%] and 3 [18%] also with no documented information regarding symptoms and suspected cause, respectively). In addition to the information from the medical history, the specific diagnostic tools used for diagnosing priapism are shown in Table 1.

**TABLE 1:** Diagnostic tools in cases presenting due to priapism (*N* = 40).

Diagnostic tools	*n* (%)
Penile blood gas analysis (BGA)	32 (80)
Sickle cell test	5 (13)
Blood screening for leukaemia	5 (13)
Angiography	5 (13)
Pelvic magnetic resonance imaging (MRI)	5 (13)
Penile duplex ultrasound	4 (10)
Haemophilia screening	4 (10)
Drug screening test	2 (5)

As based on the BGA, 29 of the 32 cases with available results were of the low‐flow type. Specific blood tests to investigate possible nondrug‐related causes of priapism gave the following results: There were five cases (13%) that tested negative for sickle cell anaemia, five cases (13%) that tested negative for leukaemia in the differential blood count and one positive case out of four cases tested for haemophilia. A drug screening test showed a positive result for amphetamines in one case and for benzodiazepines (lorazepam among the entry medication) and opiates in another case. Penile duplex ultrasound showed evidence of low arterial flow in the corpus cavernosum in two cases and no evidence of pathology in the corpus cavernosum in three cases. CT angiography showed evidence of an arteriovenous fistula in one of the five cases examined. Magnetic resonance imaging (MRI) of the pelvis showed no evidence of pathologies in any of the five cases examined (Table 1). The negative MRI result in the same patient 6 months later was supplemented by a positive result of an arteriovenous fistula in the CT angiography scan; the rest of the MRI and angiography examinations were performed in different patients. After the diagnosis of priapism, 32 cases (80%) stayed in the hospital, seven cases (18%) were discharged home (five of them with a follow‐up consultation planned) and in one case (3%) there was no documentation about the further management of the patient. The suspected causes for the priapism based on the patient history and diagnostics were idiopathic (*n* = 25, 63%), drug‐induced (*n* = 10, 25%), recreational drugs (*n* = 3, 8%), vascular (*n* = 2, 5%) and infection (*n* = 1, 3%); in five cases, two potential causes were described, while in three cases it was unclear whether the cause was idiopathic or recreational drugs. In four cases (10%), no suspected causes were documented. Four of the ten drug‐induced cases were related to a corpus cavernosum autoinjection therapy. In two cases, the presentation took place more than 20 and 32 h after the injection, while this information was not available in the other two cases. The other six suspected drug‐induced cases are described in more detail in Table 2.

**TABLE 2:** Characteristics of presentations with suspected drug‐induced priapism (excluding cases related to corpus cavernosum autoinjection therapy).

Age group (years)	Entry medication	Type of priapism	Suspected causative agent	Therapeutic interventions	Recurrence	Recurrence prophylaxis	Causality assessment (total score)∗
50–59	Urapidil	Low flow	Urapidil	Puncture of the corpus cavernosum, urapidil stopped	Unknown	No	Probable (6)
30–39	Trazodone 400 mg (single take)	Low flow	Trazodone	Puncture of the corpus cavernosum, trazodone stopped	Unknown	No	Probable (6)
50–59	Sildenafil	Low flow	Sildenafil	Puncture of the corpus cavernosum, sildenafil stopped, Winter shunt installation	No	No	Probable (5)
70–79	Sildenafil, tamsulosin, elvitegravir/tenofovir, alafenamide/elvitegravir/cobicistat, candesartan	Low flow	Sildenafil	Puncture of the corpus cavernosum, sildenafil stopped, Winter shunt installation	No	No	Probable (7)
30–39	Trazodone, amoxicillin/clavulanic acid, proton pump inhibitor	Low flow	Trazodone	Puncture of the corpus cavernosum, no documentation regarding continuation of trazodone	Yes (after 1 week)	No	Probable (6)
80–89	Trazodone, mucolytics	Low flow	Trazodone	Puncture of the corpus cavernosum, trazodone stopped, Winter shunt installation	No	No	Probable (6)

^∗^Using the Naranjo causality criteria [8]; score ≥ 9: definite, 5–8: probable, 1–4: possible, ≤ 0: doubtful adverse drug reaction.

The therapeutic measures initiated are shown in Table 3.

**TABLE 3:** Initiated therapeutic measures (*N* = 40).

	*n* (%)
Puncture of the corpus cavernosum and injection of noradrenalin and adrenalin	35 (88)
Winter shunt installation	15 (38)
Drugs	8 (20)

*Analgesics*	
*Metamizole*	5
*Paracetamol*	2
*Morphine*	2
*Pethidine*	2
*Fentanyl*	1

*PDE-5 inhibitors*	
*Sildenafil*	1
*Tadalafil*	1

*Other*	
*Amoxicillin/clavulanic acid*	4
Stopping of drugs	6 (15)
Decongestive measure (cooling)	4 (10)
Self‐regressive	2 (5)
No data	2 (5)

In five cases, a therapy was continued (tadalafil in 3 cases and metamizole and diazepam in 1 case each). In 14 out of the 15 cases, the Winter shunt was inserted after puncture of the corpus cavernosum proved to be insufficient.

Although the initial therapy was assessed as successful in 35 cases (88%), 13 cases had at least one recurrence (maximum number of recurrences during hospitalisation: 4). These recurrences occurred most commonly within one week (10 cases), while the patients were still hospitalised, and after a week following hospital discharge (3 cases). Recurrences occurred in 9 of the cases that received a Winter shunt. In one case, recurrences every few days for about half a year were described. In turn, five of these 13 cases were prescribed recurrence prophylaxis, while ten cases received recurrence prophylaxis with no recurrences observed (seven cases) or documented (three cases) during treatment. The recurrence prophylaxis consisted of one medication, with the exception of one case in which two drugs (tadalafil and diazepam) were given. Drugs prescribed as recurrence prophylaxis in the medical records (*n* = 15) were tadalafil (*n* = 9; among those 4 [27%] with documented and 5 [33%] with no documented or observed recurrences), diazepam (*n* = 4; all four with no documented or observed recurrences) and metamizole (*n* = 3), although for the latter it was not clear based on the documentation whether the prophylaxis was for priapism or pain recurrence.

## 4. Discussion

This retrospective study analysed presentations due to priapism at an urban tertiary care hospital in Switzerland. Over a period of nearly 13 years, there were approximately three presentations per year that could be included, while 19 additional cases could not be included due to lack of consent for data use. The typical patient was middle‐aged, presenting via the emergency department with ischaemic priapism and was hospitalised for further management. Most cases were described as idiopathic, while drugs were the second most commonly suspected cause. As a therapeutic measure, puncture of the corpus cavernosum and injection of sympathomimetics were undertaken in the majority of the cases, while drug treatment to prevent recurrence was only initiated in a few cases. These findings are in line with the recent guidelines of the European Association of Urology (EAU) [5], according to which the most common causes of ischaemic priapism are idiopathic. Other commonly reported causative factors are sickle cell disease, haematological dyscrasias, neoplastic syndromes and pharmacological agents [5]. The guidelines for first‐line treatment of priapism include medical management (e.g., aspiration and intracavernous injection of a sympathomimetic agent) [5].

Ischaemic (low‐flow) priapism is a medical emergency, due to the risk of permanent erectile dysfunction, which increases with longer duration of the event [9]. It is therefore very important to achieve timely diagnosis, especially to distinguish between ischaemic and nonischaemic priapism, and this must be followed by initiation of therapeutic measures. Besides the history (e.g., a trauma preceding the nonischaemic priapism), some clinical characteristics may be of help in distinguishing the two forms. In low‐flow priapism, the penis is typically painful, rigid, erect and tender, with dark blood on corporal aspiration, while in high‐flow priapism, the penis is less painful, not fully rigid, nontender, and the blood is bright red on corporal aspiration [6, 7]. A BGA is usually performed as a further diagnostic tool (also in the majority of our cases). Further possible diagnostics include specific laboratory and toxicology tests if a disease (e.g., sickle cell disease, haemophilia or blood malignancy) or recreational drugs are suspected as a cause (also in our case series performed only in 2 cases and not routinely), but such evaluations should not delay the initiation of therapy in acute cases [7]. In our study, all tested cases were negative for sickle cell anaemia and leukaemia, and there was one positive case for haemophilia (priapism cause described as ‘idiopathic’ in this case). A duplex ultrasound of the penis (also performed in some of our cases) can be used instead of the BGA as a diagnostic tool to differentiate between ischaemic and nonischaemic cases if a BGA is not available and the examiner is sufficiently skilled and may also show anatomical abnormalities (e.g., arterial fistula) [6], while other diagnostic tests such as radiologic imaging are less relevant in the context of the acute management of priapism cases [7].

For low‐flow priapism, first‐line emergency management includes intracavernosal treatments with alpha‐adrenergic agents. According to guidelines, phenylephrine is the injectable agent of preference, due to its rapid onset, short duration of action and alpha‐1 selectivity, and better outcomes are reported when combined with aspiration and saline irrigation [5, 7]. Other sympathomimetic agents are mentioned as alternatives in European guidelines [5] and were used in our case series, possibly because of limited availability of phenylephrine in the past and/or cost‐related factors. If these measures fail, a distal corporoglanular shunt should be performed [7]. Also, in our case series, a shunt was not employed as first‐line therapy, but only when puncture of the corpus cavernosum was insufficient. For cases with no improvement despite a shunt or after ischaemic priapism for more than 36 h, a penile prosthesis can be discussed if the patient still wishes to be able to perform sexual intercourse despite fibrosis of the corpora cavernosa [7]. Nonischaemic priapism on the other hand is not a medical emergency and treatment can be less aggressive, with an outpatient period of observation and diagnostic testing (e.g., Doppler ultrasound) and treatment planning (e.g., embolisation) as a further step [10].

Medication groups that have been associated with priapism include neuroleptics (approximately one‐third to half of the drug‐induced cases), antidepressants (especially trazodone), α‐receptor blockers (∼9%), antihypertensives, propofol, testosterone, anticoagulants and substances that promote erection [1, 5, 7]. In the case of neuroleptics, priapism can occur after the first dose or after long‐term treatment [4], with postulated greater risk for substances with higher alpha‐1 receptor affinity [3, 11]. Interestingly, although drugs used as treatment for erectile dysfunction would theoretically be expected to be among the drug‐induced causes for priapism, as based on studies using adverse reports to the U.S. Food and Drug Administration (FDA), second‐generation neuroleptics and trazodone were 2.6 and 2.0 times, respectively, more commonly reported than PDE‐5 inhibitors [12]. Moreover, in a more recent evaluation of the FDA’s pharmacovigilance database [13], drugs described as high‐risk group included trazodone, olanzapine and tadalafil, while other second‐generation neuroleptics were also described as triggers. These findings were also confirmed in a retrospective cohort analysis study using a database of insurance claims [14]. Despite neuroleptics being reported in the literature as the most common group in drug‐induced priapism cases, there were no such cases in our study. However, in three of the included cases, the antidepressant trazodone was the suspected agent. The mechanism by which trazodone leads to priapism is thought to be related to its alpha‐adrenoceptor blocking properties [15]. Nevertheless, when a drug is suspected as a cause of priapism, it should be discontinued, and an agent with no or less alpha‐antagonistic effects can be evaluated as an alternative if needed [1]. Importantly, patients need to be informed about this possible adverse drug effect when starting a therapy with such an agent, and tolerance and adherence should regularly be discussed, since some patients might not address it timely due to feelings of shame.

Among drugs used as recurrence prophylaxis, tadalafil was the most commonly prescribed in our cases. Reports of the (apparently paradoxical) use of PDE‐5 inhibitors as prophylaxis include a case of a patient with stuttering idiopathic priapism, who was prescribed tadalafil daily for 6 months without further recurrences during 24 months of monitoring [16], a patient with sickle cell disease receiving sildenafil for longer than one year [17], a retrospective case series of patients with stuttering or idiopathic priapism receiving sildenafil or tadalafil [18], a report of daily tadalafil administration to seven patients with recurrent priapism with a mean follow‐up of 6 months [19] and a retrospective analysis of 24 patients with recurrent ischaemic priapism that indicated a reduction of emergency department visits for ischaemic priapism under regimented PDE‐5 treatment (sildenafil 25 mg daily or tadalafil 5–10 mg 3 times weekly) [20]. This paradoxical use of PDE‐5 inhibitors is also described in current guidelines [5], with their use in low doses exhibiting preventive effects in idiopathic and stuttering priapism, despite priapism due to the use of PDE‐5 inhibitors having been observed in individual cases. However, in some of these cases, further risk factors for priapism were present and it is unclear whether PDE‐5 inhibitors per se can cause ischaemic priapism [5]. A postulated mechanism for this effect is that priapism might be associated with dysregulation of the endothelial nitric oxide synthase signalling pathway, which might be controlled by PDE‐5 inhibitors [20–22]. Other drugs discussed in the literature as recurrence prophylaxis include antiandrogens [23], baclofen [24], botulinum neurotoxin injections [25] and ketoconazole with prednisone [26].

Limitations of our study include its retrospective design, with missing data (e.g., erection duration not documented in almost half of the cases, with 1/3 of those also missing information regarding specific symptoms, which might be due to lower quality of documentation by some physicians and/or lack of time in an emergency setting) and lack of standardised data collection, the fact that 19 cases could not be included due to lack of consent, that data from a single urban tertiary centre might not be representative for other populations and that management supplies (e.g., availability of phenylephrine) may vary in other hospitals and emergency departments. These constraints limit the external validity of the study and emphasise the need for multicentre validation. Furthermore, some information used for the causality assessment was also missing in some cases (e.g., question regarding previous similar reaction: 2 cases clearly documented as first occurrence; in the other cases no explicit information available, therefore 0 points [‘*do not know*’] were given), which might have led to lower causality ratings in some cases. However, despite this limitation, the causality score ranged between 5 and 7, corresponding to ‘probable’ in all assessed cases. The strengths of our study include the identification of cases using search terms instead of diagnosis codes, the individual review of the cases by one of the authors and the detailed collection of data, including potential causes as well as diagnostic and therapeutic measures.

## 5. Conclusion

In conclusion, presentations due to priapism appear to be rare, but the majority of the cases presented with low‐flow (ischaemic) priapism, which is a medical emergency. In most cases, no specific cause could be identified (cases described as ‘idiopathic’). Drugs were the second most common suspected cause, with corpus cavernosum autoinjection therapy, the antidepressant trazodone and the PDE‐5 inhibitor sildenafil being reported as suspected agents in most cases. At the same time, the PDE‐5 inhibitor tadalafil was prescribed as recurrence prophylaxis in some cases (off‐label use). In contrast to other reports in the literature, neuroleptics were not among the commonly suspected drug causes. These findings can be used to identify areas requiring further research (e.g., drugs used as recurrence prophylaxis) and raise awareness of this potentially severe medical complication that patients are often too ashamed to mention.

## Funding

This project received no external funding. Open access publishing facilitated by Inselspital Universitatsspital Bern, as part of the Wiley ‐ Inselspital Universitatsspital Bern agreement via the Consortium Of Swiss Academic Libraries.

## Conflicts of Interest

The authors declare no conflicts of interest.

## Data Availability

Not publicly available, but data supporting the findings of the study are available within the article.
